# Multiplex structural variant detection by whole-genome mapping and nanopore sequencing

**DOI:** 10.1038/s41598-022-10483-7

**Published:** 2022-04-20

**Authors:** Lahari Uppuluri, Yilin Wang, Eleanor Young, Jessica S. Wong, Heba Z. Abid, Ming Xiao

**Affiliations:** 1 School of Biomedical Engineering, Science and Health Systems, Drexel University, Philadelphia, PA USA; 2 Department of Mechanical Engineering and Mechanics, Drexel University, Philadelphia, PA USA; 3 Center for Genomic Sciences, Institute of Molecular Medicine and Infectious Disease, Drexel University, Philadelphia, PA USA

**Keywords:** Genomic analysis, Interspersed repetitive sequences, Structural variation, DNA sequencing, Nanopores

## Abstract

Identification of structural variants (SVs) breakpoints is important in studying mutations, mutagenic causes, and functional impacts. Next-generation sequencing and whole-genome optical mapping are extensively used in SV discovery and characterization. However, multiple platforms and computational approaches are needed for comprehensive analysis, making it resource-intensive and expensive. Here, we propose a strategy combining optical mapping and cas9-assisted targeted nanopore sequencing to analyze SVs. Optical mapping can economically and quickly detect SVs across a whole genome but does not provide sequence-level information or precisely resolve breakpoints. Furthermore, since only a subset of all SVs is known to affect biology, we attempted to type a subset of all SVs using targeted nanopore sequencing. Using our approach, we resolved the breakpoints of five deletions, five insertions, and an inversion, in a single experiment.

## Introduction

Genomic structural variations (SVs) are associated with complex and multifactorial disorders^1–3^. The discovery of SVs is complicated by the variations in their sizes (ranging from 50 bps to several Mbs) and the variety in classifications (deletions, insertions, translocations, inversions, and copy number variations)^4^. These factors severely restrict the employment of a single technology or approach for SV investigations. For instance, short-read high throughput sequencing technologies were widely used to investigate and resolve smaller SVs^5^. They are highly accurate but short reads are less suited for long and complex SV characterizations^6^. Recent long-read sequencing technologies have the advantage of longer reads lengths for investigating large SVs and but suffer from low throughput, high error rates, and are prohibitively expensive^7–9^.

Single-molecule optical mapping SV detection technology, with its raw molecule length averaging 300 kbp, enables cheaper and quicker SV characterization and is widely adopted for creating maps of long-range SVs and complex rearrangements including repeats or segmental duplications^10, 11^. But it lacks sequence-level resolution and using optical mapping alone, it is not possible to accurately pinpoint SV breakpoints.

To comprehensively perform SV discovery and analysis, a combination of multi-platform and computational approaches are being increasingly employed^12–17^. These approaches maximize haplotype-resolved SV discovery and rare SV characterization but are expensive, resource-intensive, and add complexity to data analysis, limiting their adoption for routine SV characterization or large-scale or population studies and emphasizing the need for accessible methodologies for SV discovery, validation, and genotyping. A higher and stringent burden is placed on genome analysis methodologies for SV discovery. Therefore, multiplatform approaches are needed to circumvent the earlier mentioned technological limitations of any single genome analysis technology. However, post discovery, it is more economical to characterize SVs extensively and accurately for breakpoint detection, genotyping, SNP detection, or copy number variations, using next-generation sequencing^4, 18, 19^.

Several reports have been published where optical mapping was used to detect SVs (1 kbp—several Mbp) including some complex cases^11, 15, 20, 21^. Although the precise location of the SV breakpoints and sequence information is lacking, the SV region obtained from optical mapping can be leveraged subsequently for targeted sequencing. On the other hand, several targeted sequencing methodologies have also been reported for validation and genotyping of SVs^18, 22–24^. Here, we present an SV typing methodology combining optical mapping and cas9-assisted targeted long-read sequencing. As a proof of concept, in this paper, we demonstrate the application of our methodology in resolving fourteen different SVs that were detected by optical mapping. Following our approach, we could precisely pinpoint the breakpoints of six deletions, seven insertions, and an inversion. Our universal and flexible methodology can be employed in targeting multiple loci in one or more samples efficiently and economically. The simplicity of the associated analysis can benefit accelerated SV discovery and typing screens for routine diagnostics and association studies.

## Results

High throughput optical mapping creates a motif-labels-based map of the whole genome. Based on the spacing between labels, label gain or label loss, SVs (deletions, inversions, insertions, translocations, array expansions/contractions, duplications, and translocations) are detected. We generated DLE labeled whole-genome assembly and detected 2280 SVs of 2 kbp or longer in NA12878, including 1265 insertions, 759 deletions, and 256 inversions. However, whole-genome mapping cannot pinpoint the exact breakpoints, which are important for diagnostics and target association studies. As only a subset of the SVs may affect biology, we devised a strategy that relies on whole-genome optical mapping for SV discovery and nanopore sequencing for resolving specific SV loci to locate the exact breakpoints.

In the proof-of-concept experiment, we designed a set of sgRNAs to target five deletions, five insertions, and an inversion to validate and localize the breakpoints with our cas9-assisted targeted nanopore sequencing. For each SV, we designed a unique gRNA pair that was used in a cas9-mediated targeted fragmentation reaction. We synthesized a single sgRNA mix for all deletions and insertions and used it in a single-tube cas9-cleavage reaction to generate the target fragments. After fragmentation reaction, all targets were amplified and sequenced on a single nanopore flongle.

**Figure 1:**
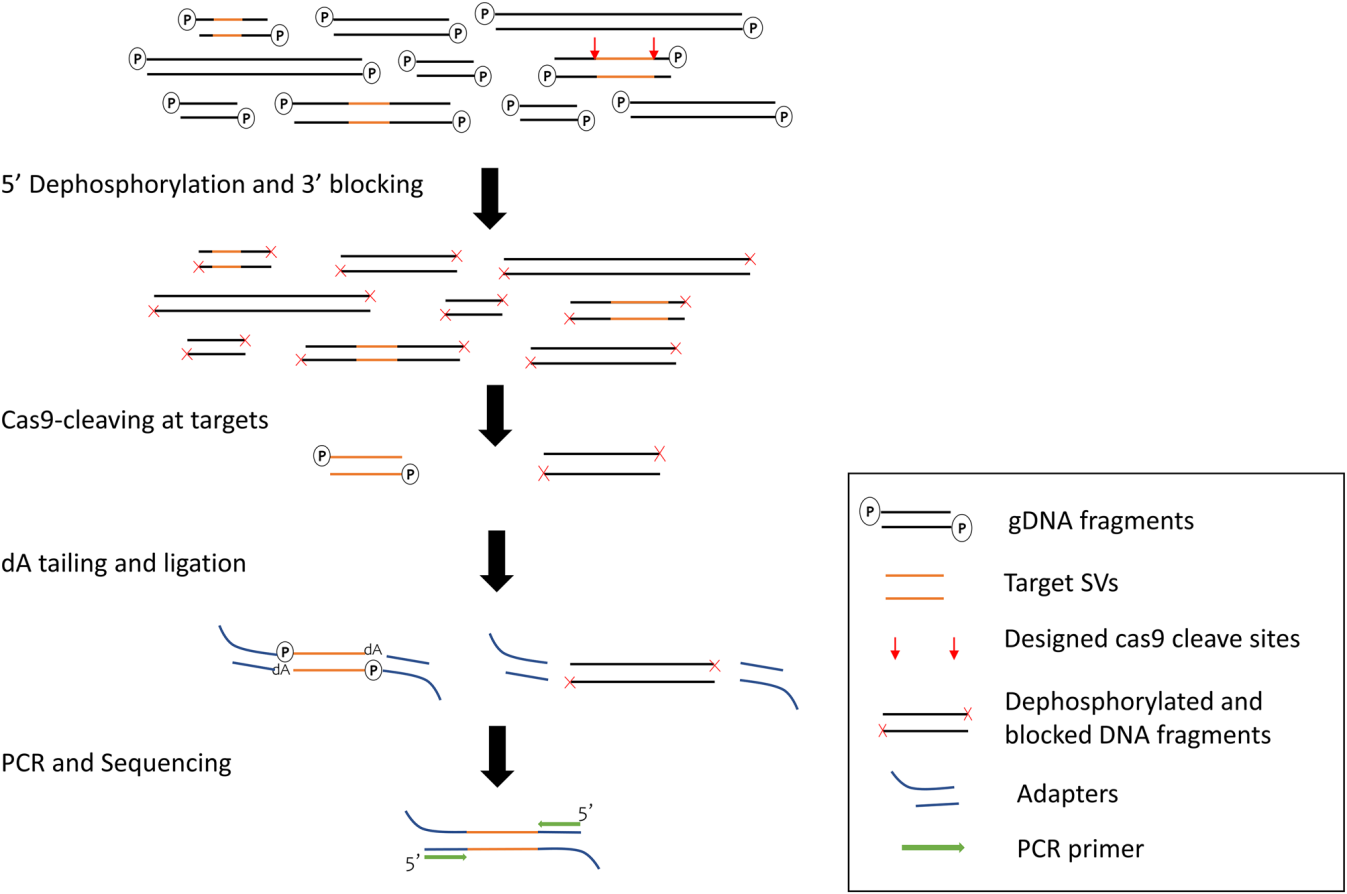
Schematic showing cas9-assisted targeted nanopore sequencing workflow. Genomic DNA was blocked at 5′ and 3′ ends to discourage non-specific ligation. The target loci were cut at the designed cleave sites there by exposing fresh DNA ends amenable to a universal adapter ligation. Following this, all loci were PCR amplified. Purified amplicons were sequenced on a nanopore flongle.

To efficiently generate the 11 target fragments, which is a tiny fraction (~ 0.03%) of the whole genome, we optimized our protocol to suppress non-target fragments^25^. Two blocking steps were performed as shown in Fig. 1. Here, the DNA fragments are shown as black bars with target sites as orange bars and the designed gRNA sites as red arrows. In the first blocking step, the 3′ ends at internal nick and break sites were blocked by incorporating dideoxynucleotides. In the subsequent step, the 5′ ends at internal nick and break sites were also blocked by dephosphorylation to discourage non-specific dA-tailing and adapter ligation. The adapter-ligated fragments were PCR amplified, purified, and then sequenced on as nanopore flongle, generating a median coverage of 17× at target sites. Using our approach, we precisely detected the breakpoints at base-level resolution for five deletions, five insertions, and an inversion (Table S1, Supplementary Information).

**Figure 2:**
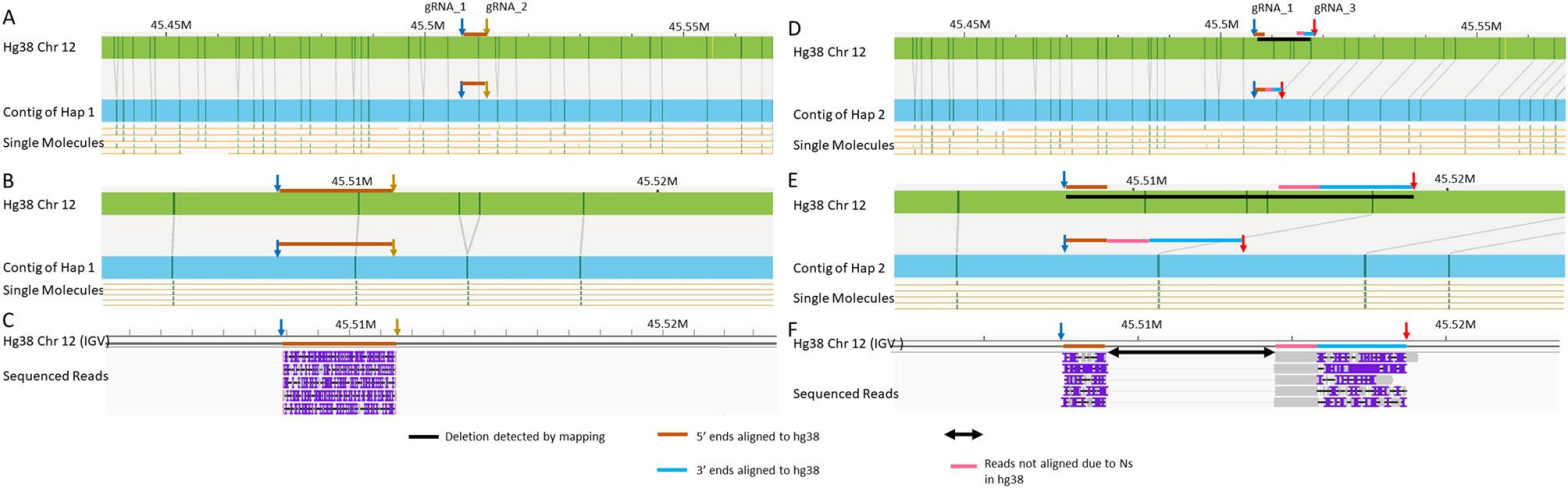
A heterozygous deletion detected with optical mapping resolved with our cas9-assisted targeted nanopore sequencing approach. (**A,D**) Show the two haplotype contigs of chromosome 12 between 45.45 and 45.55 Mbp. (**A**) Has no deletion while (**D**) shows a deletion detected by optical mapping, represented as black bar. (**B**) Shows the zoomed in view of the non-deletion haplotype contig around 45.51 Mbp. The blue and yellow arrows here represent the gRNAs designed to target this region on this haplotype. The brown bar represents the fragment generated by the gRNA pair. (**C**) Shows the sequenced reads of expected length aligning between the gRNA cut sites as predicted. Coverage was 171×. (**E**) Shows the zoomed in view of the deletion haplotype contig around 45.51 Mbp. The blue and red arrows here represent the gRNAs designed to target the deletion containing region. The three colored bars brown, pink and blue together represent the fragment generated by the gRNA pair. (**F**) Shows the sequenced reads aligning between the gRNA cut sites with a gap in between as expected. Coverage was 5×. The brown bar represents the part of fragments aligning on the 5′ end and the blue bar represents the part of fragments aligning on the 3′ end. The pink bar represents the part of fragments that do not align to hg38.

The sequencing result of a deletion is shown in Fig. 2. This is a heterozygous deletion of 13.2 kbp on chromosome 12 between 45,504,427 and 45,517,614 detected by optical mapping. Figure 2A,D show the mapping results of the two haplotypes found at this location in NA12878. While the contig of the haplotype 1 has the same pattern as the hg38 reference with no detected deletion; a deletion of 13.2kbp was detected between 45,504,427 to 45,517,614 bp on the contig of haplotype 2 (marked with a horizontal black bar). Several motifs on hg38 were missing on the contig of haplotype 2.

To differentiate this heterozygous deletion, we designed three gRNAs, acting as two pairs. The first pair is shown in Fig. 2B (a zoomed-in view of Fig. 2A) is indicated as two arrows (blue and yellow). The gRNA pair on this haplotype was designed to generate a 3.5kbp fragment of the same length as hg38 (represented by a brown bar) for sequencing.

For the deletion-containing haplotype 2, earlier mentioned gRNA_1 (blue arrow) at 45507940 bp was reused along with gRNA_3 (red arrow) at 45518921 bp. As seen in Fig. 2D, the gRNA pair is situated about 11kbp apart on hg38 and spans the entire deletion length (black bar) that was detected on hg38 and extends into known deletion-flanking regions. After the cas9 cutting, the gRNA pair was expected to generate a shorter fragment of unknown length but containing at least a part of the deletion-flanking sequences and the deletion breakpoints. In Fig. 2E, these sequences can be seen in between the two gRNA as a three-segment bar comprising of the brown segment at the 5’ end, followed by a pink segment in the middle and a blue segment at the 3’ end.

Two sets of reads were observed, one set of reads, 3.5kbp long was aligned between 45,507,952 and 45,511,479 bp flanked by gRNA_1 and gRNA_2 (Fig. 2C), which matched the expected fragment length. These reads confirm the presence of undeleted haplotype as hg38. The second set of the reads was about ~ 5.5 kbp and was aligned between 45,507,952–45,518,937 bp (Fig. 2F). The aligned reads were discontinuous with a portion aligning at the 5’ end and at 3’ ends respectively with a gap (black arrow) in between. The reads aligning at the 5’ end (brown bar) start at the expected gRNA_1 cut site and are ~ 1.36 kbp in length. The reads aligning at the 3’ end (pink and blue bars) is ~ 4.1kbp long and end at the expected cut site of gRNA_3. The black arrow in the middle is the true deleted sequence detected with the breakpoint at 45,509,371 bp. The brown and blue bar segments aligned well to hg38 but the reads at the pink bar were soft clipped and showed poor alignment due to the presence of Ns on hg38 at this location. The consensus sequence of the pink bar-segment reads shows the sequences that are not present in hg38. Clearly, our sequencing result validated our approach and detected a heterozygous deletion.

**Figure 3:**
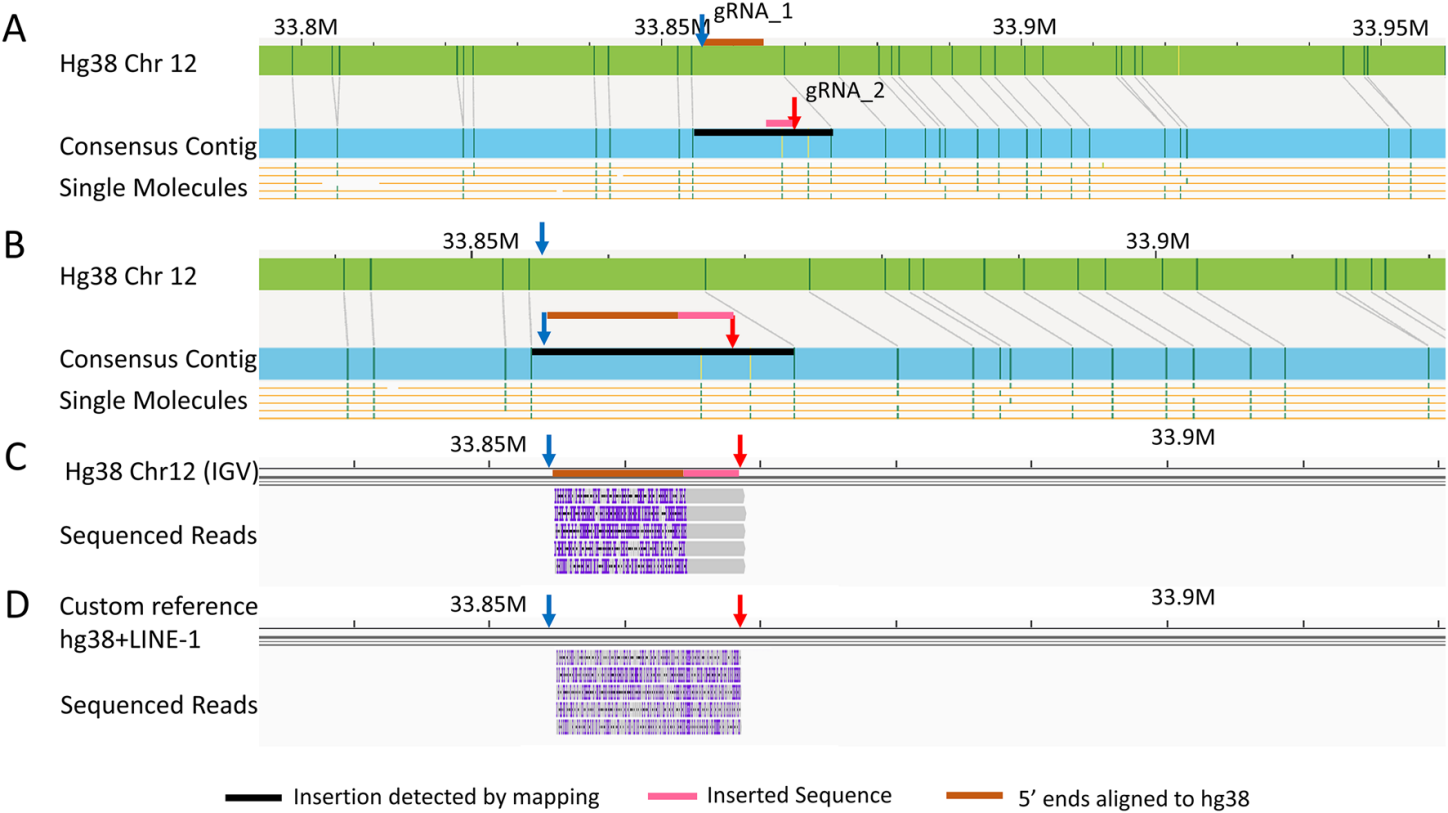
An insertion detected with optical mapping resolved with our cas9-assisted targeted nanopore sequencing approach. (**A**) Shows a black bar at the location where the insertion was detected on chromosome 12 contig by optical mapping. (**B**) Shows a zoomed in view the insertion on the contig and the gRNA pair, blue and red arrows, designed to target this region. The brown and pink bars represent the expected fragment generated by the gRNA pair. The brown bar represents part of fragment aligning to hg38 and the pink bar represents part of fragment containing partial insertion sequence. (**C**) Shows the sequenced reads aligning between the designed gRNA cut sites. Coverage was 5X. All fragments had two segments as expected. The part of reads representing aligning portions are marked by a brown bar on the IGV reference. The part of reads not aligning are marked by the pink bar. (**D**) Shows the same reads aligning to a custom reference where the reference consisted of hg38 sequence with a LINE-1 reference inserted at the detected breakpoint.

We also targeted multiple long interspersed nuclear elements (LINE-1) insertions in the same reaction. The optical mapping data of one example, a homozygous insertion on chromosome 12 between 33,854,180 and 33,867,084 bp, is shown in Fig. 3. In Fig. 3A, the estimated insertion region is marked with a black bar, and the locus spans about 12.9 kbp on the contig. In Fig. 3B (a zoomed-in view of Fig. 3A at this location), two additional labels (vertical yellow ticks) were observed on the consensus contigs as compared to the hg38. Based on the extra-label patterns (yellow ticks), and the insertion size, this insertion was suspected to be a LINE-1 insertion. To resolve the breakpoint, we designed two gRNA to target it; gRNA_1 (blue arrow) was expected to cut at 33,854,858 bp on hg38. The other gRNA, (gRNA_2, red arrow) was designed based on GenBank L1.2/L19088 reference and was expected to cut only within LINE-1 insertions. The gRNA pair was expected to generate a fragment of ~ 15 kbp length (see Fig. 3B) comprising a partial insertion-flanking sequence (brown bar), breakpoint, and a partial inserted sequence (pink bar). As expected, the reads from this SV region were found to align starting from the gRNA_1 cut site and extending into the insertion region. (Fig. 3B) The sequenced reads aligned to hg38 starting from gRNA_1 cut site (33,864,471 bp), were 8.8kbp long and observed to have two segments represented by a brown bar and a pink bar. The sequences under the brown bar aligned well to hg38 between 33,864,471–33,864,402 bp and the sequences under the pink bar did not align to hg38. But the pink segment was aligned perfectly with the putative LINE-1 reference (GenBank L1.2/L19088) as shown in Fig. 3D. The breakpoint of insertion in between the brown and pink bar, at 33,864,403 bp. The insertion sequence was validated by aligning the reads to a custom reference created with a LINE-1 insertion from the detected breakpoint. Further, the fragments ended at the gRNA_2 cut site (red arrow) inside the insertion (Fig. 3D).

**Figure 4:**
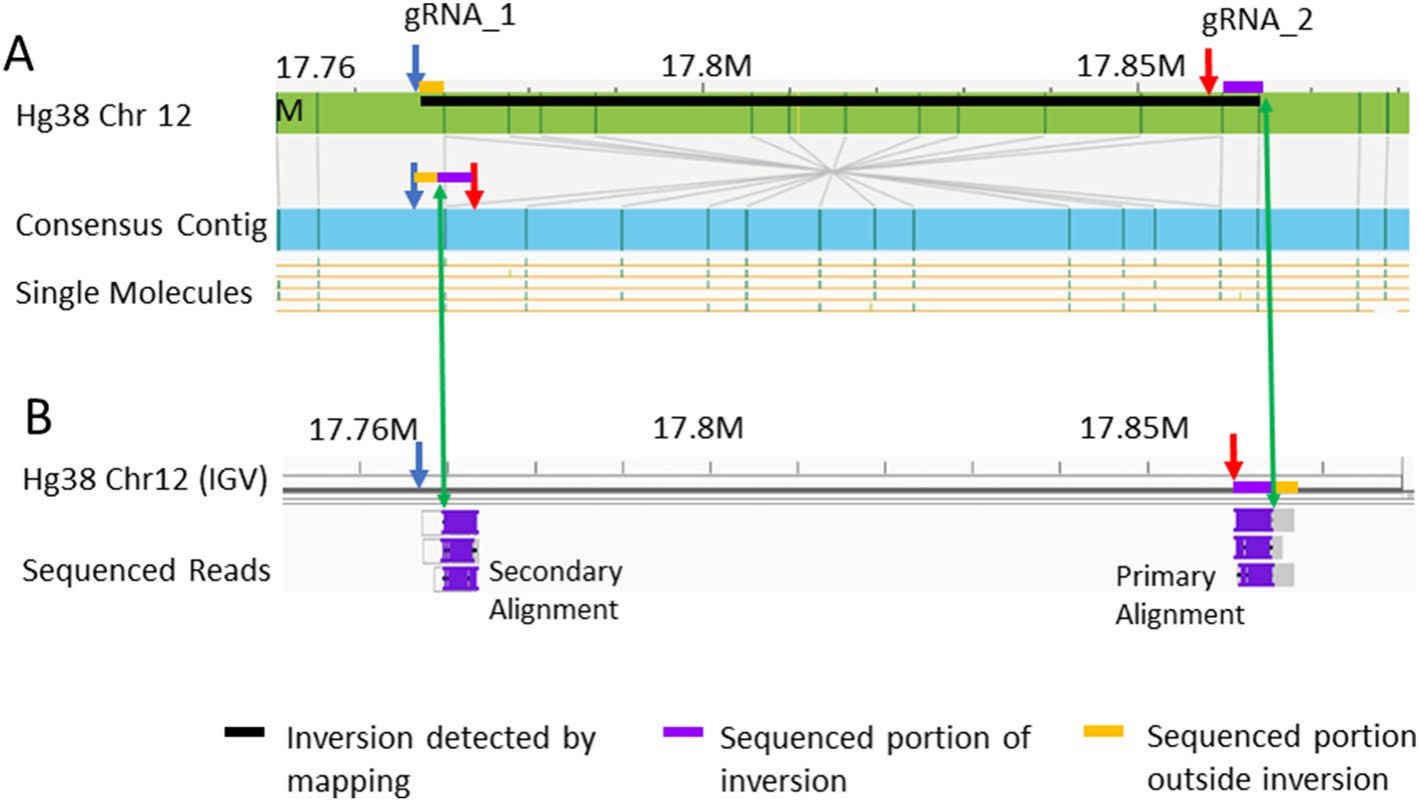
An inversion detected with optical mapping resolved with our cas9-assisted targeted nanopore sequencing approach. (**A**) Shows a black bar at the location where the inversion was detected on chromosome 12 contig by optical mapping. The blue and red arrows represent the gRNA pair designed to target this region. This gRNA pair is expected to generate a fragment represented by the yellow-purple bars. The yellow bar represents the part of fragment flanking the inversion while the purple represents the part of fragment inside the inversion. (**B**) Shows a set of sequenced reads aligning to this region. Coverage was 6×. They had distinct aligning and non-aligning segments as expected represented by the purple and yellow bars on the IGV reference respectively. The reads had a primarily alignment to hg38 on the right side of the inversion as expected in 3′ to 5′ orientation starting from the one designed gRNA cut site. The same reads showed a secondary alignment on the left side of inversion with an inverted orientation as expected also from a designed cut site.

Finally, we applied our strategy to resolve the breakpoints of a homozygous inversion on chromosome 12. Optical mapping estimated the inversion to be ~ 90 kbp from 17,770,250 to 17,859,681 bp with several labels in this region mapping in the 3′ to  > 5′ orientation (Fig. 4A). To resolve this inversion, two gRNA were designed with one in the inversion-flanking region at 11,176,068 bp (gRNA_1, blue arrow) and one inside the inversion at 17857700 bp (gRNA_2, red arrow). Due to the inversion in the sample, gRNA_2 is expected to move towards the 5′ end, closer to gRNA_1, and generate a ~ 6.5 kbp fragment. The fragments are predicted to have a part of the inverted region (purple bar) and a part of the inversion-flanking region (yellow bar) as shown in Fig. 4A. However, since there are two different match locations, these fragments are expected to align to hg38 starting at gRNA_1 or gRNA_2 cut site and extend into the flanking region. The 6.5kbp long sequenced fragments aligned the SV region are shown in Fig. 4B (on the right), aligning from gRNA_2 at 17,857,721 bp to 17864360 bp in 5′ to  3′ orientation. This set of reads aligned partially to hg38, denoted by a purple bar (17,857,721–17,861,570 bp), followed by a non-aligning part denoted by a yellow bar (17,861,571–17,864,360 bp). This is as expected since the yellow bar represents part of the reads aligning to the inversion flanking region starting at the blue arrow on hg38 in 5′ to 3′ direction. In Fig. 4B on the left, the same set of reads can also be seen aligning to hg38 near the other breakpoint from 17,772,217 bp in 3′ to 5′ orientation. We reason that this is due to the high sequence similarity within the 5 kbp span extending into the inversion from both predicted breakpoints. Taken together, we detected both breakpoints of this inversion—the first at 17,768,358 bp and the second one at 17,861,570 bp, shown by green arrows in Fig. 4B.

## Discussion

SV discovery is burdened by stringent thresholds against false positives and technological limitations. Resource-intensive multi-platform and computational approaches are needed for comprehensive SV characterization and discourage widespread adoption^19^. However, only a subset of the discovered have biological implications. Targeting only a few SVs for characterization would ease the economic and computational burden. To efficiently target and resolve multiple SVs, we presented a simple strategy in this paper leveraging whole-genome mapping and cas9-assisted targeted nanopore sequencing.

Optical mapping technology creates whole-genome maps based on motif-specific labeling. Based on the label density and distribution, diverse SVs > 1 kbp can be more economically detected^15^. SV characterization with optical mapping is cheaper and quicker but provides only the SV region. Using just optical mapping, it is not feasible to accurately resolve breakpoints. However, leveraging the information from optical mapping, it is possible to design gRNA for cas9-assisted targeted sequencing of SV loci making it more economically feasible to characterize multiple SVs in multiple samples, all at the same time.

We demonstrated the application of our strategy to resolve the breakpoints of fourteen SVs, including six deletions, seven insertions, and an inversion as a proof of concept (Table S1, Supplementary Information). In our experiments, we designed 24 unique gRNA to target and cleave at each SV loci (Supplementary Information). We adapted our sample preparation and performed 5′ and 3′ end-inactivation, attempting to suppress the likelihood of sequencing adapter ligation to non-target fragments. We detected both haplotypes in a heterozygous deletion in chromosome 12 and resolve the SV breakpoint on the deletion-containing haplotype. Insertions are a difficult class of SVs to characterize using conventional methods due to the new unknown sequence involved^4, 19^. Whole-genome sequencing can address this problem, but the huge amount of sequencing data needed along with the associated low throughput and high error rates makes it impractical and discourage technology adoption for this purpose. Targeted sequencing in conjunction with optical mapping can potentially address the gaps by enriching the insertion-containing loci. With our approach, we were able to detect a breakpoint of LINE-1 insertions, which are thought to be variably inserted among individuals and are associated with multiple genetic conditions and various types of cancers^26^. Nevertheless, our gRNA design for LINE-1 insertions is based on a known reference sequence. For cases where prior knowledge of inserted sequence is lacking or the exact locations of deleted or inserted segments are not clear, the gRNAs can be designed to cut the DNA in the flanking regions of SVs, limiting the sizes of those that can be targeted using our approach. We targeted three additional SVs, two insertions, and a deletion, by designing probes in the flanking regions. The results from two of these SVs are presented in Figs. S3 and S4 in Supplementary Information. Table S1 (Supplementary Information) contains the genomic regions, probe sequences used, and the resolved breakpoints for the three additional SVs). Finally, the detection and analysis of inversions with conventional NGS tools are complicated as they tend to be heavily populated with long and highly similar segmental duplications^14^. We sought to overcome this problem by designing a pair of probes in such a way that one probe is in the inversion-flanking region and the other one is inside the inversion. We could successfully resolve both breakpoints of a homozygous inversion on chromosome 12.

Most recently, Beyter et al. performed a large-scale population study and used sequencing data alone for SV discovery and analysis by using Oxford Nanopore’s PromethION flowcell^27^. As of today, the cost to acquire PromethION flowcells was $5600 for 4 flow cells ($1400 per flow cell) according to the oxford nanopore website. Here, we present an alternative approach to analyzing SVs by incorporating optical maps and cas9-assisted targeted nanopore sequencing. Our approach allows for flexible and multiplex SV analyses at a competitive cost. For our experiment, we obtained a wide range of SV information by performing DLE-based optical mapping on the sample for < $500. Synthesis of sgRNA costs significantly lower than directly purchasing the RNA. So, we synthesized the sgRNA required for the experiment in a one-pot transcription reaction for < $5. We sequenced our sample on a flongle (~ $70), which also lessened the genomic DNA input burden. Recently, our group reported the synthesis and use of up to 200 synthesized gRNA in a single reaction further expanding the assay’s applicability in SV analysis^28^. Our approach relies on PCR to counteract low data yield on a flongle. Performing PCR enrichment makes the assay more economical, but target sizes are limited to under 25–30 kbp. It is also possible to follow our approach without PCR to analyze larger SVs. However, without PCR enrichment, one would need more genomic input DNA and more amount of data to achieve similar coverages at target loci, adding to cost. For more complex SVs, multi-color whole-genome mapping can be used to create custom maps of the sample and validate the combinations of gRNA to be used in an experiment^20, 29, 30^. Using our universal approach, it is possible to target multiple SVs in a sample or analyze an SV across multiple samples, enabling precise detection of breakpoints economically.

## Materials and methods

### Single guide RNA synthesis

55-base oligomers encoded with T7 promoter sequence (5′-TTCTAATACGACTCACTATAG-3′), 20-base target sequence, and an overlap sequence (5′-GTTTTAGAGCTAGA-3′) were designed and purchased from IDT. An 80 base long overlap-complementary sequence (5′-AAAGCACCGACTCGGTGCCACTTTTTCAAGTTGATAACGGACTAGCCTTATTTTAACTTGCTATTTCTAGCTCTAAAAC-3′) was also purchased from IDT. Briefly, 10 µM of equimolar pooled oligomers and 10 µM of overlap-complementary overlap sequence containing oligomer are first mixed, denatured at 95 °C for 15 s, and later allowed to hybridize at 43 °C for 5 min in 1× NEBuffer 2.0 (NEB). The hybridized oligos were then extended with 5U of Klenow exo- at 37 °C for 1 h in the presence of 2 mM dNTPs. Next, an exonuclease treatment was carried out at 37 °C for 1 h with 10U of Exonuclease I (NEB) in 1× Exonuclease buffer (NEB). The dsDNA was purified with a Qiagen Nucleotide removal kit and quantified via absorbance spectroscopy before use in RNA synthesis. Using the above dsDNA, a single guide RNA mixture (sgRNA) was synthesized following the manufacturer’s recommendations (HiScribe T7 High Yield RNA Synthesis Kit, NEB). After transcription and DNaseI (NEB) treatment, the sgRNA was purified using spin columns (Monarch RNA Cleanup Kit T2030, NEB) and quantified via absorbance spectroscopy before use. The 260/280 ratio for dsDNA was ~ 1.8–1.9 and the synthesized sgRNA was ~ 2.0.

### Sample preparation

First, a 3’ blocking step is performed where 1000 ng of high molecular weight NA12878 DNA (Coriell Institute) was incubated in a tube with 5U of Klenow exo- (NEB) and 1× 3.1 buffer (NEB) in the presence of 10 µM ddNTPs at 37 °C for 1 h. Next, 3U of Shrimp Alkaline Phosphatase is added to the tube and incubated at 37 °C for 1 h and then at 65 °C for 15 min.

In a separate tube, cas9-sgRNA complex was made by incubating 400 ng of cas9 Nuclease (NEB) in 1× NEBuffer 3.1 (NEB) and 2.5 µM sgRNA at 37 °C for 15 min. Then, the cas9-sgRNA Complex was added to the SAP treated DNA and incubated at 37 °C for 2 h. After the cas9 digestion step, a dA-tailing step was performed by adding 200 µM dATP and 5U of Taq DNA Polymerase (NEB) to the reaction and incubating at 72 °C for 5 min. The reaction was purified then with AMPURE XP beads before proceeding to the next step where a universal Y-adapter was ligated to the dA-tailed fragments using NEB Quick Ligation Kit. The reaction was purified using 0.4× AMPURE XP beads and subsequently, 10 ng DNA was carried into PCR reactions. PCR was set up following the manufacturer’s recommendations for NEB LongAmp Taq DNA polymerase.

PCR amplicons were purified and pooled for sequencing library preparation with SQK-LSK109 Ligation Sequencing Kit 1D, Oxford Nanopore Technologies (ONT) following manufacturer’s recommendations.

### Sequencing and data analysis

Sequencing was carried out on a FLO-FLG001 flongle flow cell (ONT). The experiment was set up and run and live base calling was performed using MinKNOW 20.1.3, Guppy 4.2.2 software. The fastq files generated after completion of the reads were combined and aligned to the hg38 reference using minimap2 with default settings^31^. Integrated Genomics Viewer (IGV 2.12.2) was used to visualize the assembled contigs aligned to reference. The reads aligned in the deletion and insertion SV regions were shortlisted and analyzed further for each locus. The reads in the inversion region were aligned to an alternative reference of 150kbp encompassing the inversion locus. We were able to resolve the breakpoints for the SVs presented in this manuscript using minimap2 and IGV alone. For more complex cases, other aligners or tools might be needed.

### Bionano genome mapping data and assembly

NA12878 sample was labeled as per manufacturer’s recommendations using Bionano DLS chemistry. The labeled DNA was loaded onto Bionano Genomics Saphyr Chip G1.2 and imaged on Saphyr system to collect 480 Gbp data. The single molecules image data was de novo assembled into optical genome maps using Bionano Solve with default settings. The assembly was visualized on Bionano Access webserver.

## Supplementary Information

Supplementary Information.

## Data Availability

The sequencing read data as well as the alignment files can be accessed via NCBI under BioProject PRJNA823696 [https://www.ncbi.nlm.nih.gov/bioproject/823696].
