# Supplementary material for: Multiplex structural variant detection by whole-genome mapping and nanopore sequencing
Source: Sci Rep. 2022 Apr 20;12:6512. doi: 10.1038/s41598-022-10483-7 (PMC9021263; doi:10.1038/s41598-022-10483-7)
Supplement: Supplementary file 1 — Supplementary Information. [file 41598_2022_10483_MOESM1_ESM.pdf]

Multiplex structural variant detection by whole-genome mapping and nanopore sequencing.

Lahari Uppuluri [1,2], Yilin Wang[1], Eleanor Young[1], Jessica S. Wong[1], Heba Z. Abid[1], and Ming Xiao[1,3*]

[1] School of Biomedical Engineering, Science and Health Systems, Drexel University, Philadelphia, Pennsylvania, United States

[2] Department of Mechanical Engineering and Mechanics, Drexel University, Philadelphia, Pennsylvania, United States

[3] Center for Genomic Sciences, Institute of Molecular Medicine and Infectious Disease, Drexel University, Philadelphia, Pennsylvania, United States

*Corresponding author

Email: ming.xiao@drexel.edu (MX)

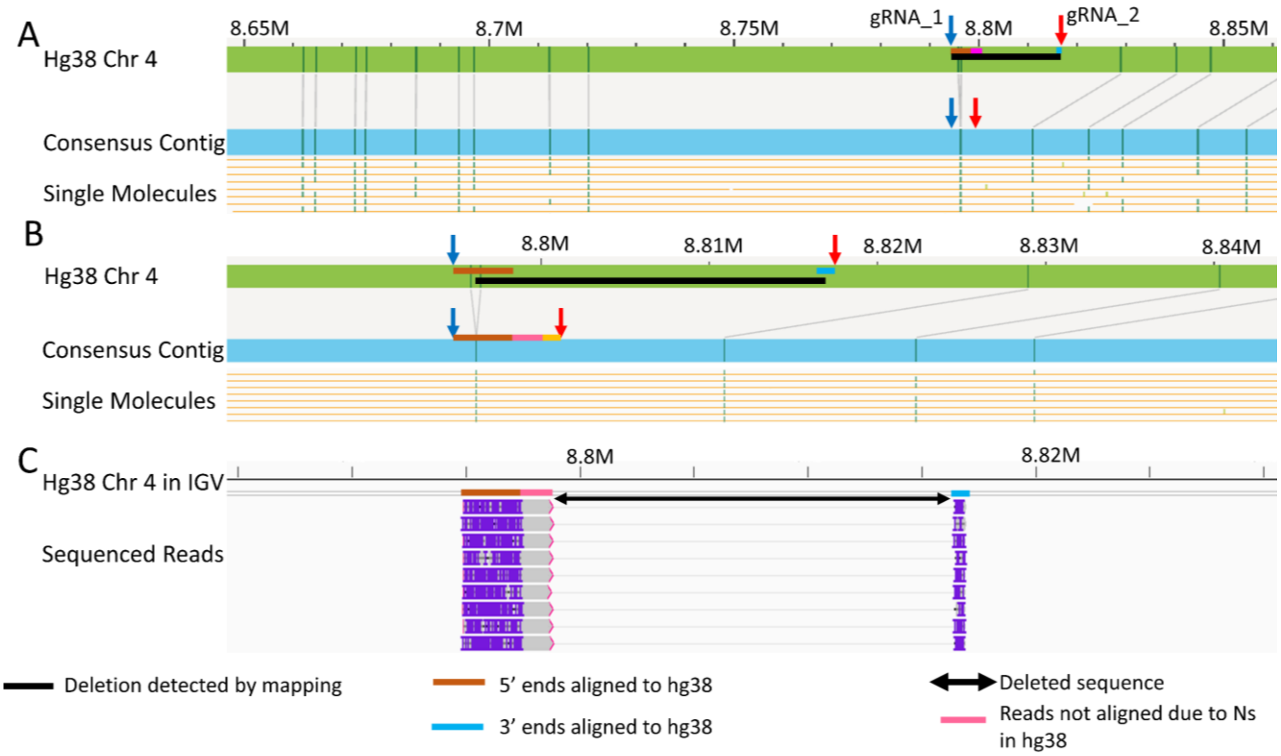

Figure S1. A homozygous deletion at chr4:8796117-8828957 resolved with our cas9-assisted targeted nanopore sequencing approach. Panel A shows optical map contigs of chromosome 4 between 8.65 and 8.85 Mbp. B shows a zoomed-in view of the maps around the deletion indicated by black bar. The blue and red arrows here represent the gRNAs designed to target the presumed deletion containing region. The three colored bars brown, pink and blue together represent the fragment generated by the gRNA pair. Panel C shows the sequenced reads aligning between the gRNA cut sites with a gap in between as expected. Coverage was 9X. The brown bar represents the part of fragments aligning on the 5'end and the blue bar represents the part of fragments aligning on the 3' end. The pink bar represents the part of fragments that do not align to hg38.

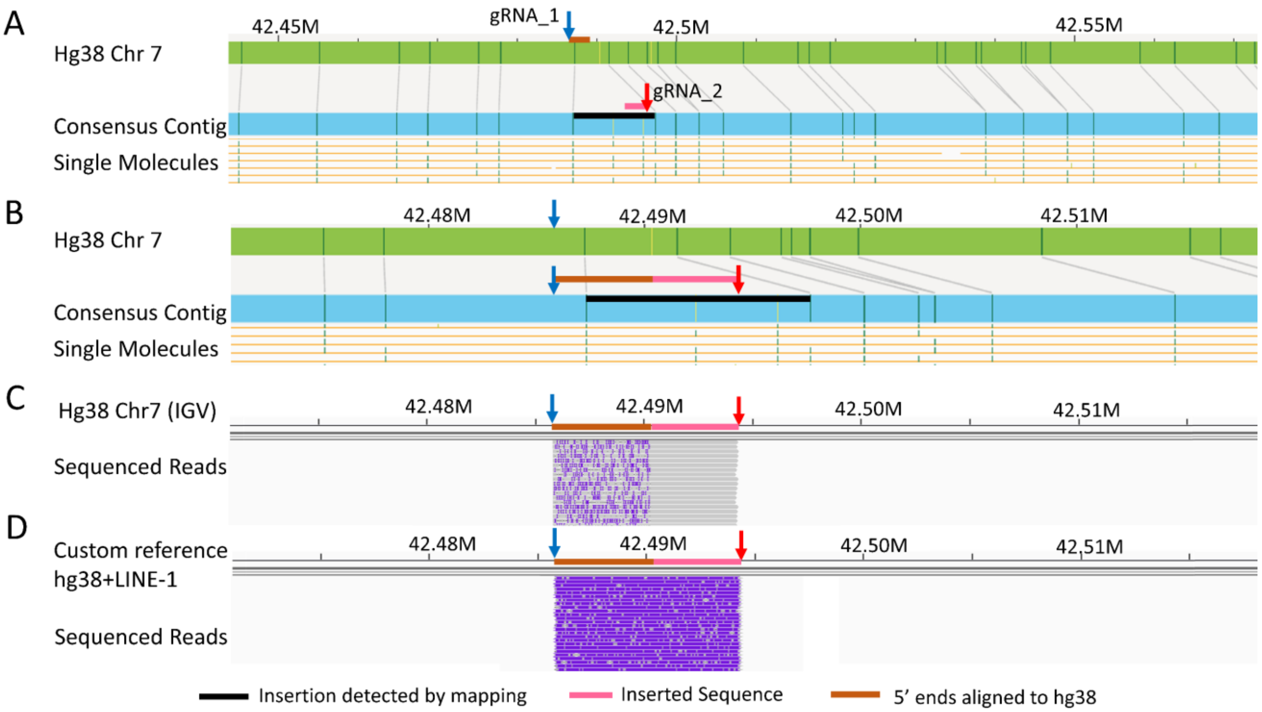

Figure S2. A LINE-1 insertion at chr7:42487230-42491515 resolved with our cas9-assisted targeted nanopore sequencing approach. Panel A shows optical map contigs of chromosome 7 between 42.45 and 42.55 Mbp. B shows a zoomed-in view of the maps around the insertion, indicated by black bar. The blue and red arrows here represent the gRNAs designed to target the presumed deletion containing region. The two, colored bars brown and pink together represent the fragment generated by the gRNA pair. Panel C shows the sequenced reads aligning between the gRNA cut sites expected. Coverage was 79X. The brown bar represents the part of fragments aligning on the 5'end and the blue bar represents the part of fragments aligning on the 3' end. The pink bar represents reads not aligning to hg38. Panel D shows the same reads aligning to a custom reference where the reference consisted of hg38 sequence with a LINE-1 reference inserted at the detected breakpoint.

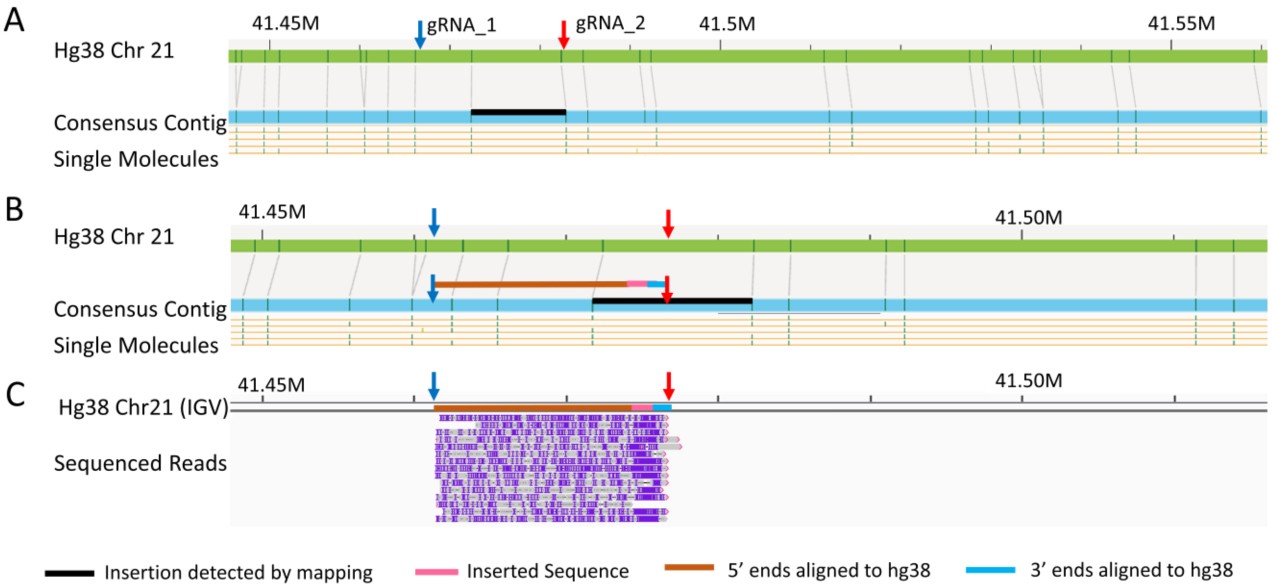

Figure S3. A homozygous insertion at chr21:41454955-41484741 resolved with our cas9-assisted targeted nanopore sequencing approach. Panel A shows optical map contigs of chromosome 21 between 41.45 and 41.55 Mbp. B shows a zoomed-in view of the maps around the insertion indicated by black bar. The blue and red arrows here represent the gRNAs designed to target the insertion containing and its flanking regions. The three colored bars brown, pink and blue together represent the fragment generated by the gRNA pair. Panel C shows the sequenced reads aligning between the expected gRNA cut sites with an insertion marked. Coverage was 14X. The brown bar represents the part of fragments aligning on the 5'end and the blue bar represents the part of fragments aligning on the 3' end. The pink bar represents the part of fragments that do not align to hg38.

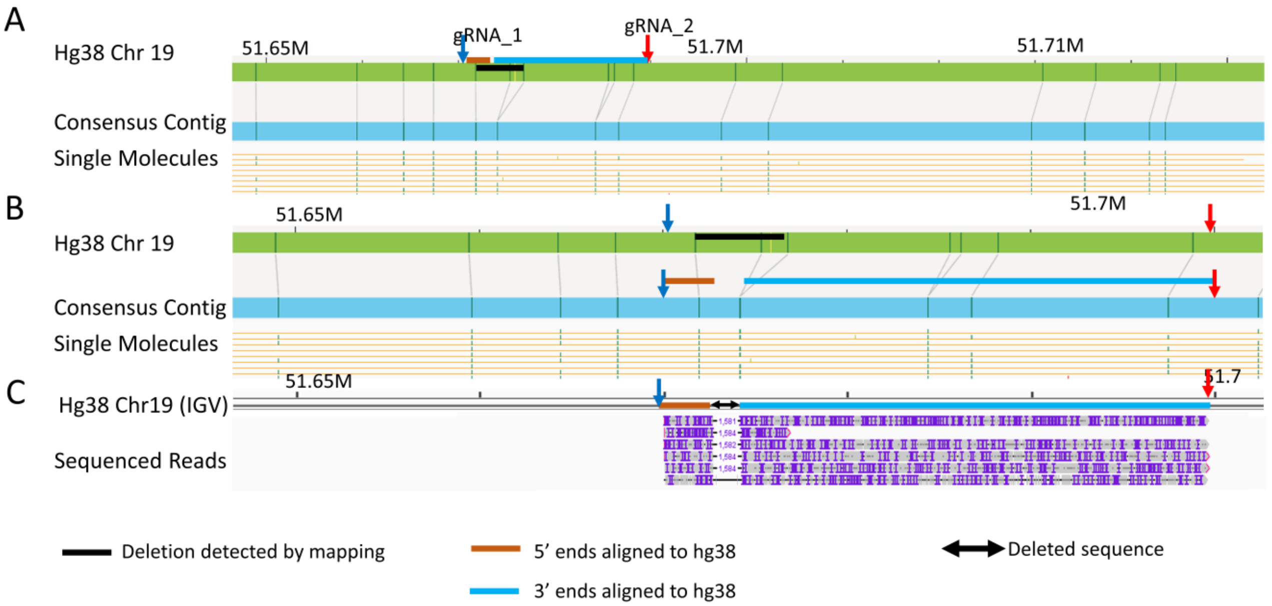

Figure S4. A deletion at chr19:51651518-51757499 resolved with our cas9-assisted targeted nanopore sequencing approach. Panel A shows optical map contigs of chromosome 4 between 51.65 and 51.72 Mbp. B shows a zoomed-in view of the maps around the deletion indicated by black bar. The blue and red arrows here represent the gRNAs designed to target the presumed deletion containing region. The two, colored bars brown and blue together represent the fragment generated by the gRNA pair. Panel C shows the sequenced reads aligning between the gRNA cut sites with a gap in between as expected. Coverage was 6X. The brown bar represents the part of fragments aligning on the 5'end and the blue bar represents the part of fragments aligning on the 3' end.

Table S1: List of target SVs designed gRNAs and detected breakpoints.

SV Genomic Region	gRNA_1	gRNA_2	gRNA_3	SV	Detected breakpoints (bp)
chr4:8796117-8828957	GGGGGACTCTGAACACAAGT	GGATACGTCACCTCTTTGAA		Deletion	8797478
chr12:45504427-45517614	TTGCACCACAACTTGTCAGA	CACATTGAGAACCTCTGCTA	GTCCAAGAGTAAGCACCCCA	Deletion	45509371
chr19:8282857-8293646	GTCCCAATCTGGACTCCTAG	CATCGACTGCCCAAGCCAAG		Deletion*	8277069
chr1:152555548-152587768	ACTGACACCTCTATAGATAC	CTATTTGCATTGACCACTGC	ACTTTTCTTGGATGATACCG	Deletion	152583066
chr6:167590126-167652737	CATCTGGTCTGGTCACCTAC	AAGCCAAGGTATACCCATGC	GGTCGAATTCCGGAGACACT	Deletion	167591394
chr3:186650273-186655454	TTGACCCAAGGGTAGAACTA	AGGAAGATCTACCAAGCCAA		Insertion**	186654352
chr7:42487230-42491515	GATGAATTCGATTTCTGTGT	AGGAAGATCTACCAAGCCAA		Insertion**	42490277
chr6:13500995-13504649	AGGAAGATCTACCAAGCCAA	AAGGATACTAAAATACCCAC		Insertion**	13502803
chr1:48958322-48966134	TAGCCTTTAGCATCACACTC	AGGAAGATCTACCAAGCCAA		Insertion**	48963085
chr12:33854180-33867084	AAGTTCTTTATCTGTTTGTT	AGGAAGATCTACCAAGCCAA		Insertion**	33864403
chr12:17649268-17936894	GGGACATTACGTCAACCTAA	ATAGGAGTTTACCCAGTCCT		Inversion	17768358, 17861570
chr21:41454955-41484741	TGAGGCAGGAAAATCGAGTC	GCCCTGGCAGCCACCTAACG		Insertion	41474066
chr3:10121283-10167205	CAGGAGTTTAAGACCAGCCT	CCAGGAGTTAAAACCAGCCT		Insertion	10134067, 10134166
chr19:51651518-51757499	GATCACCTGAGGTCAGGAGT	TTGCCCAGGCTGGCGTACAG		Deletion	51672676, 51674261

* We estimate the deletion size to be ~15 kbp and that secondary alignment is due to sequence similarities in the flanking regions.
** LINE-1 Insertion
